# Real-World Observations in the Treatment of Aortic Stenosis With the Transfemoral SAPIEN 3 Transcatheter Heart Valve: Insights From China

**DOI:** 10.31083/RCM28800

**Published:** 2025-05-22

**Authors:** Jie Dong, Ziping Li, Peijian Wei, Yiming Yan, Guangzhi Zhao, Wenbin Ouyang, Shiguo Li, Yongquan Xie, Junyi Wan, Donghui Xu, Fengwen Zhang, Gejun Zhang, Shouzheng Wang, Xiangbin Pan

**Affiliations:** ^1^Department of Structural Heart Disease, National Center for Cardiovascular Disease, China & Fuwai Hospital, Chinese Academy of Medical Sciences & Peking Union Medical College, 100037 Beijing, China; ^2^National Health Commission Key Laboratory of Cardiovascular Regeneration Medicine, 100037 Beijing, China; ^3^Key Laboratory of Innovative Cardiovascular Devices, Chinese Academy of Medical Sciences, 100037 Beijing, China; ^4^National Clinical Research Center for Cardiovascular Diseases, Fuwai Hospital, Chinese Academy of Medical Sciences, 100037 Beijing, China

**Keywords:** aortic stenosis, SAPIEN 3, transcatheter aortic valve replacement, bicuspid aortic valves

## Abstract

**Background:**

Transcatheter aortic valve replacement (TAVR) has emerged as the preferred treatment for symptomatic severe aortic stenosis (AS). However, China’s unique patient population presents distinct challenges, including a higher prevalence of bicuspid aortic valves (BAVs) and severe valve calcification. This study used real-world clinical data from Chinese patients to assess the safety and efficacy of the SAPIEN 3 balloon-expandable transcatheter heart valve (THV) in TAVR, particularly in patients with BAVs.

**Methods:**

This retrospective, multicenter study enrolled consecutive severe AS patients treated with SAPIEN 3 THVs via a transfemoral approach from June 2020 to March 2024. The primary endpoint was 30-day mortality, while secondary endpoints included procedural mortality, procedural success, conversion to surgery, coronary artery occlusion, THV-in-THV deployment, permanent pacemaker implantation, and paravalvular leaks (PVLs).

**Results:**

Among the 1642 enrolled patients, 56.0% had BAVs, and 44.0% had tricuspid aortic valves (TAVs). The 30-day mortality rate was 0.90%. Propensity score matching revealed no statistically significant differences between patients with BAVs and TAVs in terms of 30-day mortality (odds ratio (OR): 1.51, 95% confidence interval (CI): 0.42 to 5.36; *p* = 0.531), immediate procedural mortality, procedural success, coronary artery occlusion, THV-in-THV deployment, permanent pacemaker implantation, or moderate to severe PVLs. However, a significant difference was found in the conversion rate to open surgery (OR: 5.07, 95% CI: 1.11 to 23.2; *p* = 0.036).

**Conclusions:**

This study demonstrates the safety and feasibility of SAPIEN 3 balloon-expandable THVs in TAVR for Chinese patients with severe AS, including those with BAV stenosis. These findings challenge historical relative contraindications for TAVR in BAV patients and highlight the potential of TAVR in diverse patient populations. Larger prospective studies with extended follow-ups are needed to refine patient selection and evaluate longer-term outcomes.

## 1. Introduction

Transcatheter aortic valve replacement (TAVR) has become the preferred therapeutic option for symptomatic severe aortic stenosis (AS) over the past decade. Numerous studies affirm the noninferiority or superiority of TAVR compared to surgical aortic valve replacement (SAVR) across various procedural risk scenarios [1, 2, 3, 4, 5, 6, 7]. Improvements in interventional techniques, procedural proficiency, and innovations in transcatheter heart valve (THV) design have significantly contributed to enhanced clinical outcomes.

In China, the reported incidence of valvular heart disease ranges from 2.5 to 3.2 per thousand, though this is likely underestimated. With over 290 million people aged 60 years or older, more than 100 medical centers in China now perform TAVR procedures for severe AS [8, 9, 10]. Comparative analysis of TAVR candidates in China and Western nations reveals several distinctions. Notably, there is a higher prevalence of bicuspid aortic valve (BAV), a greater incidence of type 0 valves, substantial aortic valve calcification, a higher frequency of aortic regurgitation (AR) relative to AS, a significant proportion of cases attributed to rheumatic causes, and narrower femoral artery diameters [11, 12]. BAV patients often exhibit oval valve annuli, trapezoidal valve leaflets, asymmetric valve structure, and pronounced calcification, leading to issues such as valve distortion, displacement, and incomplete attachment. Furthermore, TAVR in patients with irregular and severely calcified BAVs has been associated with an increased risk of short-term aortic root rupture and moderate-to-severe AR [13, 14, 15, 16, 17]. Inadequate THV expansion and sizing also raise concerns about durability and thrombosis [18]. However, it is crucial to note that most landmark randomized clinical trials thus far have excluded BAV patients [19]. Previous registries have shown comparable outcomes of TAVR between BAV and tricuspid aortic valve (TAV) patients [20, 21], but these registries were limited in detailed information regarding various BAV morphologies, which may affect the outcomes after TAVR [13].

With ongoing device innovation and the accumulation of evidence-based medical data, the scope of TAVR application has steadily expanded. In 2015, the The Food and Drug Administration (FDA) approved the SAPIEN 3 transcatheter aortic valve (Edwards Lifesciences, Irvine, CA, USA) for patients experiencing symptomatic severe AS at high risk for SAVR surgery. This device addressed issues such as paravalvular leakage (PVL) and introduced a smaller sheath and improved delivery system. In 2019, the PARTNER 3 trial results were officially published [6], providing an evidence-based foundation for TAVR utilization in low-risk patients. SAPIEN 3 has emerged as the only TAVR technology globally proven to be superior to SAVR in the primary endpoint of randomized controlled clinical trials for low-risk patients [6]. In May 2023, the National Medical Products Administration (NMPA) of China approved SAPIEN 3 for use in patients with severe symptomatic AS who are at high risk for or ineligible for open-heart surgery.

However, it is worth noting that the China TAVR consensus [11] still considers TAVR in BAV patients as a relative indication [22]. Given the current literature’s insufficient evidence, this study aims to address this significant clinical issue by evaluating the safety and efficacy of the transfemoral SAPIEN 3 balloon expandable THVs in TAVR for AS patients, especially those with BAV stenosis, based on real-world clinical data from consecutively enrolled patients in China who underwent implantation of this system.

## 2. Method

### 2.1 Study Population and Procedure

This retrospective, multi-center study was conducted from June 2020 to March 2024. We enrolled consecutive patients with symptomatic severe AS who were treated with the Edwards SAPIEN 3 Transcatheter Heart Valve and Commander delivery system via transfemoral approach across 123 medical centers in China. A list of participating centers is provided in **Supplementary Table 1**. The inclusion criteria were: (1) Severe symptomatic AS requiring aortic valve replacement, characterized by one or more of the following: aortic valve area (AVA) <0.8 cm^2^, indexed AVA <0.5 cm^2^/m^2^, mean gradient >40 mmHg, or peak aortic jet velocity >4.0 m/sec; (2) New York Heart Association (NYHA) functional class II or greater; (3) Agreement to comply with all required post-procedure follow-up visits. Exclusion criteria included: (1) Acute myocardial infarction ≤1 month before the intended treatment; (2) Congenitally unicuspid or non-calcified aortic valve; (3) Permanent implants from therapeutic invasive cardiac surgery (e.g., transcatheter edge-to-edge repair or ventricular septal defect closure) within 30 days before the index procedure (implantation of permanent pacemakers or implantable cardioverter-defibrillators was not exclusionary); (4) Renal insufficiency (creatinine >3.0 mg/dL) and/or receiving renal replacement therapy; (5) Untreated clinically significant coronary artery disease requiring revascularization; (6) Anomalous coronary artery interfering with proper valve placement; (7) Hypertrophic cardiomyopathy with or without obstruction (myocardial thickness >1.5 cm without a definite cause) or other cardiomyopathies (e.g., dilated cardiomyopathy); (8) Severe ventricular dysfunction, left ventricular ejection fraction (LVEF) <20%; (9) Life expectancy ≤12 months due to cancer, chronic liver disease, chronic kidney disease, or end-stage lung disease. Patients with quadricuspid aortic valve morphology were also excluded.

The SAPIEN 3 balloon expandable THVs and TAVR procedures have been described previously [23]. These devices were introduced via the transfemoral approach. The coplanar angle was adjusted angiographically during the procedure based on pre-procedural multidetector computed tomography (MDCT) (Lightspeed Volume CT, GE healthcare, Little Chalfont, UK). This included a comprehensive assessment spanning the aortic annulus to the aortic root, using parameters such as area, perimeter, supra-annular or intercommissural distance/area, and median, minimum, and maximum diameters to determine optimal THV size and implantation position.

Computed tomography (CT) (Somatom Definition Flash, Siemens Healthcare, Forchheim, Germany) imaging was also used to localize and quantify calcification burden using Agatston scoring or calcium volume measurements. Calcification was graded semi-quantitatively at both the annular and left ventricular outflow tract (LVOT) levels and assessed per cusp sector as follows: none: no calcification; mild: small, non-protruding calcifications; moderate: protruding calcifications (>1 mm) or extensive calcifications (>50% of cusp sector); and severe: protruding (>1 mm) and extensive (>50% of cusp sector) calcifications. Prosthesis size and inflation volume of the deployment balloon were determined according to the annulus size and other anatomical characteristics, including aortic–valvular complex calcification and aortic angiograms. To evaluate PVL, all patients underwent post-deployment aortic angiograms and echocardiography. Post-dilation was performed to reduce PVL to less than moderate (2+) [24]. Patients requiring post-dilation were categorized based on the final inflation volume of the deployment balloon. This trial adhered to the principles of the Declaration of Helsinki. The Institutional Review Board of the Chinese Academy of Medical Sciences, Fuwai Hospital, granted approval for this study (ethical approval number: 2022-1829), and all participants received a waiver of informed consent.

### 2.2 Study Endpoints

The primary endpoint was 30-day mortality. Secondary endpoints established based on the Valve Academic Research Consortium 2 (VARC-2) consensus [25], included immediate procedural mortality, procedural success, conversion to open surgery, coronary artery occlusion, THV-in-THV deployment, permanent pacemaker implantation, and PVLs. Procedural success was defined as the absence of procedural mortality, accurate placement of a single prosthetic heart valve in its appropriate anatomical position, achieving the intended performance of the prosthetic heart valve (no prosthesis-patient mismatch and a mean aortic valve gradient <20 mmHg or peak velocity <3 m/s), and no occurrence of moderate or severe prosthetic valve regurgitation or conversion to open surgery. THV-in-THV deployment was defined as repeat valve deployment during the same session due to initial deployment failure.

### 2.3 Statistical Analysis

Statistical analysis was performed using STATA 17.0 (StataCorp, College Station, TX, USA). A two-tailed *p*-value <0.05 was considered statistically significant. Continuous data with normal distribution are presented as means and standard deviations. Nonparametric continuous data are presented as medians with interquartile ranges, and categorical data are presented as numbers and percentages. The χ^2^-test was employed for comparison of categorical variables, and the Mann-Whitney U test and unpaired *t*-test were utilized for between-group comparisons of continuous variables with skewed and normal distributions, respectively.

Associations between aortic valve morphology and study outcomes were estimated using unadjusted logistic regression models, multivariable logistic regression models (adjusted by age and sex), and propensity score matching. Propensity score matching, a statistical analysis of observational data, attempts to reduce treatment assignment bias and mimic randomization by making the groups comparable concerning the control variables. For propensity score matching analyses, we performed a 1-to-1 matching for BAV vs. TAV in the total population based on the covariates age and sex. We used the Stata command “calipmatch” to perform a greedy matching algorithm with no replacement for all propensity score matching. A caliper width of 0.01, the standard deviation of the logit of the propensity score, was used for all matching. Additionally, we used the Stata command “adjrr” to estimate adjusted risk differences.

We imputed missing data using MissForest, a random forest imputation algorithm implemented in the R software, version 3.2.3 (R Foundation for Statistical Computing, Vienna, Austria). Variables with <25% missing values were imputed. The missing rates for the study variables are presented in **Supplementary Table 2**.

## 3. Results

### 3.1 Overall Baseline, Procedural and Outcomes Characteristics

Between June 2020 and March 2024, 1642 cases of de novo AS patients treated with transfemoral TAVR were included in this study. The average age was 72.5 ± 8.36 years, with a higher proportion of males (55.5%), and the mean LVEF was 56.3 ± 12.5%. Annulus aneurysm was observed in 4.7% of patients. Moderate to severe calcification of the annulus, LVOT, and leaflets was present in 10.1%, 4.8%, and 69.9% of patients, respectively. The most common prosthesis sizes were 23 mm and 26 mm, which accounted for 79.7% of the total. The prosthesis implantation depth was generally greater than in Western practices, with 17.1% of patients deploying the valve at a height of 100/0 and 42.6% at a height of 90/10 (Table 1, Ref. [26]).

**Table 1.** **Overall patients’ baseline and procedural characteristics**.

Characteristics		Total cohort	BAV cohort	TAV cohort	*p* value
		(n = 1642)	(n = 920)	(n = 722)	
Demographics					
	Age (years)	72.5 ± 8.36	70.6 ± 7.79	75.1 ± 8.40	<0.001
	Male (n, %)	911 (55.5)	548 (59.6)	363 (50.3)	<0.001
LVEF (%)*					0.290
		56.3 ± 12.5	55.8 ± 12.6	56.9 ± 12.3	
Annulus area (mm^2^)					<0.001
		460.0 (399.1, 532.0)	484.0 (422.0, 562.0)	432.0 (380.0, 492.0)	
Annulus diameter (mm)					<0.001
		38.7 ± 6.70	40.8 ± 6.94	36.1 ± 5.20	
Height of coronary artery (mm)					
	Left coronary artery	14.0 ± 3.20	14.7 ± 3.32	13.0 ± 2.69	<0.001
	Right coronary artery	16.4 ± 3.10	16.8 ± 3.24	15.8 ± 2.84	<0.001
Annulus aneurysm (n, %)					0.025
		77 (4.70)	53 (5.80)	24 (3.30)	
Annulus calcification (n, %)					<0.001
	None	1204 (73.3)	638 (69.3)	566 (78.4)	
	Mild	272 (16.6)	175 (19.0)	97 (13.4)	
	Moderate	116 (7.10)	71 (7.70)	45 (6.20)	
	Severe	50 (3.00)	36 (3.90)	14 (1.90)	
LVOT calcification (n, %)					0.035
	None	1424 (86.7)	779 (84.7)	645 (89.3)	
	Mild	139 (8.5)	91 (9.9)	48 (6.6)	
	Moderate	59 (3.6)	39 (4.2)	20 (2.8)	
	Severe	20 (1.2)	11 (1.2)	9 (1.2)	
Leaflet calcification (n, %)					<0.001
	None	197 (12.0)	105 (11.4)	92 (12.7)	
	Mild	298 (18.1)	138 (15.0)	160 (22.2)	
	Moderate	581 (35.4)	305 (33.2)	276 (38.2)	
	Severe	566 (34.5)	372 (40.4)	194 (26.9)	
Sizing of prosthesis (mm)					<0.001
	20	147 (9.00)	66 (7.20)	81 (11.2)	
	23	693 (42.2)	362 (39.3)	331 (45.8)	
	26	616 (37.5)	371 (40.3)	245 (33.9)	
	29	186 (11.3)	121 (13.2)	65 (9.00)	
Valvular deployment height (n, %)**					<0.001
	100/0	281 (17.1)	209 (22.7)	72 (10.0)	
	90/10	699 (42.6)	436 (47.4)	263 (36.4)	
	80/20	603 (36.7)	248 (27.0)	355 (49.2)	
	70/30	48 (2.90)	19 (2.10)	29 (4.00)	
	60/40	11 (0.70)	8 (0.90)	3 (0.40)	

*The LVEF statistics were based on 654 patients due to 988 cases with missing value for LVEF. **The implantation height was expressed as the percentage of the stent lying on the aortic and the ventricular sides [26]. Abbreviations: BAV, bicuspid aortic valve; LVEF, left ventricular ejection fraction; LVOT, left ventricular outflow tract; TAV, tricuspid aortic valve.

**Fig. 1.**
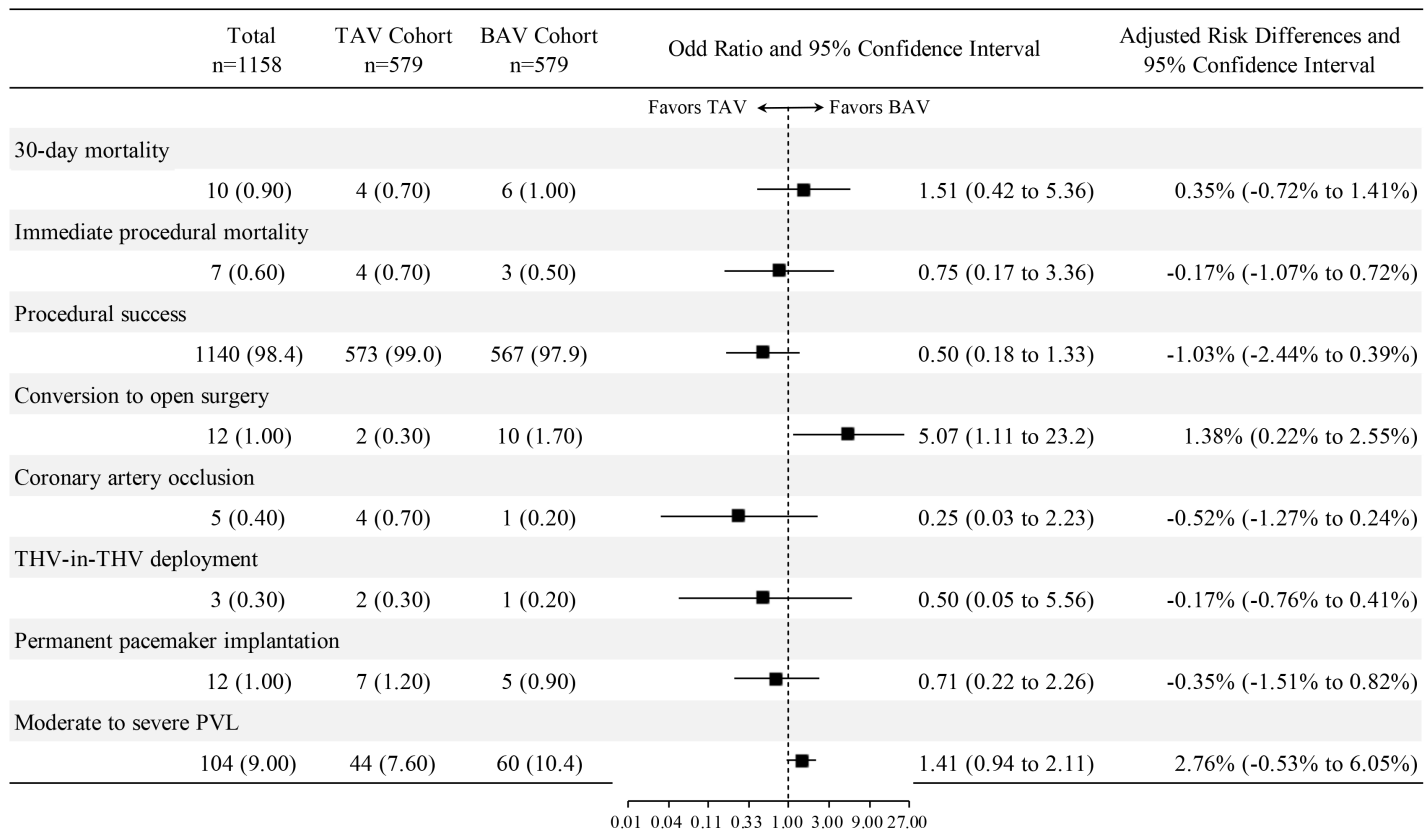
**The association between aortic valve morphology and study outcomes after propensity score matching**. Abbreviations: BAV, bicuspid aortic valve; PVL, paravalvular leak; TAV, tricuspid aortic valve; THV, transcatheter heart valve.

In terms of clinical outcomes, 30-day mortality was 0.90% (10 cases), immediate procedural mortality was 0.60% (7 cases), and the overall procedural success rate was 98.4%. Conversion to open surgery occurred in 1.00% (12 cases), coronary artery occlusion in 0.40% (5 cases), and THV-in-THV deployment age in 0.30% (3 cases). Permanent pacemaker implantation was required in 1.00% (12 patients). Specifically, 59.2% of patients had no PVL, 32.1% had mild PVL, 8.00% had moderate PVL, and 0.70% had severe PVL (Fig. 1).

### 3.2 Baseline and Procedural Characteristics Between Aortic Valve Morphologies

A comparative analysis of baseline characteristics was conducted between patients with AS who had TAV morphology (n = 722, 44.0%) and those with BAV morphology (n = 920, 56.0%) (Table 1). Patients with BAV stenosis were younger (mean age: 70.6 ± 7.79 vs. 75.1 ± 8.40 for BAV and TAV, respectively; *p* < 0.001) and more likely to be female (59.6% vs. 50.3%; *p* < 0.001). No significant difference was found in LVEF between the two groups (55.8 ± 12.6 vs. 56.9 ± 12.3 for BAV and TAV, respectively; *p* = 0.290). BAV stenosis patients had a larger annular area (484.0 [422.0 to 562.0] mm^2^ vs. 432.0 [380.0 to 492.0] mm^2^ for BAV and TAV, respectively; *p* < 0.001) and required larger prosthetic valves (*p* = 0.003). The coronary artery heights were higher in BAV stenosis patients for both the left (14.7 ± 3.32 vs. 13.0 ± 2.69; *p* < 0.001) and right (16.8 ± 3.24 vs. 15.8 ± 2.84; *p* <0.001) arteries, along with a greater burden of calcification in the annulus, LVOT, and leaflets (Table 1).

Among BAV patients, Type 1 was the most common morphology, accounting for 510 cases (55.4%), followed by Type 0 with 394 cases (42.8%), and Type 2 with 16 cases (1.73%). Compared to Type 0, Type 1 BAV stenosis patients were older (mean age: 71.5 ± 7.59 vs. 69.6 ± 7.68; *p* < 0.001), had a higher proportion of males (66.3% vs. 50.5%; *p* < 0.001), a larger annulus area (500.0 [436.0 to 576.0] mm^2^ vs. 455.5 [400.0 to 539.0] mm^2^; *p* < 0.001), and a larger aortic diameter (40.2 ± 6.98 mm vs. 41.7 ± 6.87 mm; *p* = 0.001). Type 1 patients also had lower coronary artery heights for both the left (13.6 ± 2.85 vs. 16.1 ± 3.35; *p* < 0.001) and right (16.3 ± 3.12 vs. 17.39 ± 3.30; *p* < 0.001) arteries and required larger prosthetic valves (*p* < 0.001) (**Supplementary Table 3**).

### 3.3 Associations Between Aortic Valve Morphology and Study Outcomes

Patients with BAV stenosis who underwent transfemoral TAVR did not significantly differ from those with TAV stenosis in terms of 30-day mortality (BAV vs. TAV: 0.70% vs. 0.80%, *p* = 0.770), immediate procedural mortality (BAV vs. TAV: 0.30% vs. 0.70%, *p* = 0.310), procedural success (BAV vs. TAV: 98.5% vs. 99.0%, *p* = 0.380), conversion to open surgery (BAV vs. TAV: 1.20% vs. 0.40%, *p* = 0.110), coronary artery occlusion (BAV vs. TAV: 0.20% vs. 0.60%, *p* = 0.410), THV-in-THV deployment (BAV vs. TAV: 0.20% vs. 0.40%, *p* = 0.270), and permanent pacemaker implantation (BAV vs. TAV: 1.00% vs. 1.70%, *p* = 0.270). The incidence of moderate to severe PVL (BAV vs. TAV: 9.60% vs. 7.60%, *p* = 0.160) did not significantly differ between BAV and TAV patients (Table 2). Unadjusted statistical associations are shown in **Supplementary Fig. 1**.

**Table 2.** **Overall patients’ outcomes**.

		Total cohort	BAV cohort	TAV cohort	*p* value
		(n = 1642)	(n = 920)	(n = 722)	
30-day mortality (n, %)		12 (0.70)	6 (0.70)	6 (0.80)	0.770
Immediate procedural mortality (n, %)		8 (0.50)	3 (0.30)	5 (0.70)	0.310
Procedural success (n, %)		1621 (98.7)	906 (98.5)	715 (99.0)	0.380
Conversion to open surgery (n, %)		14 (0.90)	11 (1.20)	3 (0.40)	0.110
Coronary artery occlusion (n, %)		6 (0.40)	2 (0.20)	4 (0.60)	0.410
THV-in-THV deployment (n, %)		5 (0.30)	2 (0.20)	3 (0.40)	0.660
Permanent pacemaker implantation (n, %)		21 (1.30)	9 (1.00)	12 (1.70)	0.270
PVL (n, %)					0.092
	Absent	972 (59.2)	522 (56.7)	450 (62.3)	
	Mild	527 (32.1)	310 (33.7)	217 (30.1)	
	Moderate	131 (8.00)	79 (8.60)	52 (7.20)	
	Severe	12 (0.70)	9 (1.00)	3 (0.40)	

Abbreviations: BAV, bicuspid aortic valve; PVL, paravalvular leak; TAV, tricuspid aortic valve; THV, transcatheter heart valve.

Propensity score matching created a cohort of 579 BAV stenosis patients and 579 TAV stenosis patients (80.2% of the total TAV stenosis cohort) with well-balanced demographics (Table 3). No statistically significant differences were found between BAV and TAV stenosis patients in terms of 30-day mortality [odds ratio (OR): 1.51, 95% confidence interval (CI): 0.42 to 5.36, *p* = 0.531], immediate procedural mortality (OR: 0.75, 95% CI: 0.17 to 3.36, *p* = 0.708), procedural success (OR: 0.50, 95% CI: 0.18 to 1.33, *p* = 0.165), coronary artery occlusion (OR: 0.25, 95% CI: 0.03 to 2.23, *p* = 0.215), THV-in-THV deployment (OR: 0.50, 95% CI: 0.05 to 5.56, *p* = 0.574), permanent pacemaker implantation (OR: 0.71, 95% CI: 0.22 to 2.26, *p* = 0.562), and moderate to severe PVL (OR: 1.41, 95% CI: 0.94 to 2.11, *p* = 0.098). A significant difference was found regarding conversion to open surgery (OR: 5.07, 95% CI: 1.11 to 23.2, *p* = 0.036) (Fig. 1). These findings are consistent with multivariable logistic regression models adjusted for age and sex (**Supplementary Fig. 2**).

**Table 3.** **Patients’ demographics characteristics after propensity score matching**.

Characteristics	Total cohort	BAV cohort	TAV cohort	*p* value
	(n = 1158)	(n = 579)	(n = 579)	
Age (years)	73.0 ± 7.49	73.0 ± 7.47	73.1 ± 7.51	0.890
Male (n, %)	631 (54.5)	312 (53.9)	319 (55.1)	0.720

Abbreviations: BAV, bicuspid aortic valve; TAV, tricuspid aortic valve.

## 4. Discussion

This study is a pioneering multi-center, real-world investigation aimed at assessing the safety and efficacy of SAPIEN 3 balloon-expandable THVs in TAVR for AS patients in China. Our findings strongly support the safety and feasibility of this prosthesis in challenging BAV anatomies, resulting in excellent valve performance and a notably low incidence of significant PVL. These results challenge existing contraindications and relative indications for TAVR, underscoring the potential of this approach in diverse patient populations as the field of TAVR continues to evolve.

Over the past decade, there has been a remarkable surge in the adoption of TAVR, leading to a paradigm shift in the management of severe AS [27, 28, 29]. In China, where the population is aging, there is a growing burden of degenerative valvular diseases, including AS. Additionally, Chinese patients seeking TAVR often exhibit a higher frequency of bicuspid valve morphology and more severe leaflet calcification [12], presenting unique challenges. Although TAVR was introduced later in China compared to other regions, the procedure has witnessed rapid development in recent years [30, 31]. Being the first and only approved balloon-expandable TAVR valve system in China, the SAPIEN 3 THVs have accumulated substantial clinical data and experience over a 3-year period. Our research findings provide significant insights into the efficacy and safety of SAPIEN 3 balloon-expandable THVs in treating AS across the Chinese population.

BAV anatomy can present unique challenges for TAVR, even with advances in THV design and increased operator experience, especially in Chinese AS patients with a high proportion of bicuspid valves. Historically, BAV has been considered a relative contraindication for TAVR, and these patients were excluded from major randomized trials comparing TAVR with SAVR [14]. In propensity score–matched results from the early Bicuspid AS TAVR multicenter registry, TAVR in patients with BAV stenosis was associated with a higher frequency of adverse procedural events compared to those with TAV stenosis [32, 33]. However, these differences were mainly observed in patients treated with early-generation devices, and no significant differences in procedural complications were noted when using new-generation devices [20]. In a continuous series of multicenter studies, the new-generation Lotus™ Valve System (Boston Scientific, Natick, MA, USA) demonstrated safety and feasibility in treating AS patients with BAV anatomies, resulting in excellent valve performance and a low incidence of significant PVL. However, it is important to note that this conclusion is based on products that have been withdrawn from the market and a relatively small sample size [34]. Notably, balloon-expandable THVs may offer specific advantages by providing a greater opening force to ensure circular expansion and minimize PVL [14].

Our study indicates no significant differences in 30-day mortality, procedural mortality, procedural success, coronary artery occlusion, THV-in-THV deployment, and permanent pacemaker implantation between patients with BAV stenosis and TAV stenosis who underwent TAVR with transfemoral SAPIEN 3 balloon-expandable THVs. Additionally, the incidence of moderate PVL did not significantly differ between BAV and TAV patients. While the Sievers’ classification, which considers the number and location of raphe, is commonly used to characterize various BAV morphologies [35], the successful outcomes of TAVR may be more dependent on factors such as overall calcium burden and the presence of calcified raphe, which can hinder optimal device expansion [13]. In our cohort of BAV stenosis patients, the proportion of patients with (Type 1) or without raphe (Type 0) was similar, and nearly half of the patients (40.4%) exhibited severe calcification of the leaflets.

The higher conversion to open-heart surgery rate observed in BAV stenosis patients in this cohort is consistent with results from the Society of Thoracic Surgeons–American College of Cardiology Transcatheter Valve Therapy Registry (STS/ACC TVT Registry), which confirmed a higher rate (BAV vs. TAV: 0.90% vs. 0.40%, *p* = 0.03) [36]. BAV anatomy often involves larger dimensions of all components of the aortic valve complex, a nontubular (flared or tapered) shape, more extensive calcification, the presence of a calcified raphe, heterogeneous calcium distribution, and asymmetrical morphology of the aortic valve complex and coronary anomalies, which can be procedural challenges for TAVR [14]. The newest generation of THVs could help to further improve short-term outcomes in patients with BAV stenosis to more closely match those of TAV [14].

This study has several important inherent limitations that merit acknowledgment. Given its retrospective nature, the analysis is susceptible to potential unmeasured confounders. Furthermore, it exclusively focuses on patients who underwent the TAVR procedure, which may introduce selection bias, necessitating caution when interpreting the results. Additionally, BAV anatomy is highly heterogeneous, with patients exhibiting variable degrees of valve calcification, including raphe calcification when present. Consequently, the findings of this study should not be extrapolated to the entire BAV population [37]. The anatomical risk of TAVR in BAV AS should be carefully evaluated [13]. Moreover, the relatively short follow-up duration may not adequately capture long-term outcomes, particularly concerning durability and thrombosis. Finally, many baseline characteristics, such as cardiovascular risk factors, detailed CT measurements, and comorbidities like peripheral artery disease (PAD) and chronic kidney disease (CKD), were not assessed. To gain more comprehensive insights into the safety and efficacy of TAVR in BAV patients, future research should consider larger prospective studies with extended follow-up periods.

## 5. Conclusions

In conclusion, our study contributes to the growing body of evidence surrounding TAVR, within the Chinese population. It addresses crucial clinical questions and provides valuable insights into the safety and efficacy of the transfemoral SAPIEN 3 balloon-expandable THVs, particularly in the context of BAV stenosis, offering reference for clinicians and researchers in this rapidly evolving field. Further research and ongoing evaluation of TAVR technologies and techniques are essential to refine patient selection, optimize procedural outcomes, and enhance the management of AS in diverse patient populations.

## Availability of Data and Materials

The original data provided by Edward Lifescience. The code used for data analysis can be obtained by contacting authors.

## Supplementary Material

Supplementary material associated with this article can be found, in the online version, at https://doi.org/10.31083/RCM28800.
