# Supplementary material for: Real-World Observations in the Treatment of Aortic Stenosis With the Transfemoral SAPIEN 3 Transcatheter Heart Valve: Insights From China
Source: Rev Cardiovasc Med. 2025 May 22;26(5):28800. doi: 10.31083/RCM28800 (PMC12135656; doi:10.31083/RCM28800)
Supplement: Supplementary file 1 [file 2153-8174-26-5-28800-s1.zip › Supplementary Material-V2-final.docx]

**Supplemental Material**

*Real-World Observations in the Treatment of Aortic Stenosis with the Transfemoral SAPIEN 3 Transcatheter Heart Valve: Insights from China*

**List of Content**

**Supplemental Figures**

- **Supplemental Figure 1.** The association between aortic valve morphologies and study outcomes with unadjusted logistic regression models.
- **Supplemental Figure 2.** The association between aortic valve morphologies and study outcomes adjusted by age and sex with multivariable logistic regression models.

**Supplemental Tables**

- - **Supplemental Table 1**. Missing rates of study variables and management of missingness.
  - **Supplemental Table 2.** Patients’ baseline and procedural characteristics across BAV morphologies.
  - **Supplemental Table 3.** List of included medical centers.

**Supplemental Fig. 1.** The association between aortic valve morphologies and study outcomes with unadjusted logistic regression models.

Abbreviations: BAV, bicuspid aortic valve; PVL, paravalvular leak; TAV, tricuspid aortic valve; THV, transcatheter heart valve.

**Supplemental Fig. 2.** The association between aortic valve morphologies and study outcomes adjusted by age and sex with multivariable logistic regression models.

Abbreviations: BAV, bicuspid aortic valve; PVL, paravalvular leak; TAV, tricuspid aortic valve; THV, transcatheter heart valve.

**Supplemental Table 1.** List of included medical centers.

| ID | Hospitals |
| --- | --- |
| 1 | The First Affiliated Hospital of Sun Yat-sen University |
| 2 | The Fifth Affiliated Hospital of Sun Yat-sen University |
| 3 | Ankang Central Hospital |
| 4 | Beijing Anzhen Hospital |
| 5 | Beijing Chaoyang Hospital |
| 6 | Beijing Friendship Hospital |
| 7 | Beijing Fuwai Hospital |
| 8 | The Third Hospital of Peking University (Beijing U No.3 Hospital) |
| 9 | Peking University People's Hospital (Beijing U People's Hospital) |
| 10 | Peking University Shougang Hospital (Beijing U Shougang Hospital) |
| 11 | The First Affiliated Hospital of Bengbu Medical University (Bengbu Medical U No.1 Hospital) |
| 12 | Changzhou Wujin People's Hospital |
| 13 | The First Affiliated Hospital of China Medical University |
| 14 | China-Japan Friendship Hospital |
| 15 | Chinese People's Liberation Army General Hospital (301 Hospital) |
| 16 | Chongqing Xinqiao Hospital |
| 17 | Dalian Central Hospital |
| 18 | The First Affiliated Hospital of Dalian Medical University |
| 19 | Dazhou Central Hospital |
| 20 | Dong'a County People's Hospital |
| 21 | Foshan Nanhai District People's Hospital |
| 22 | Foshan Nanhai District Second People's Hospital |
| 23 | Fujian Medical University Union Hospital |
| 24 | Fujian Provincial Hospital |
| 25 | Guangdong Provincial People's Hospital |
| 26 | Guangdong Provincial Hospital of Traditional Chinese Medicine |
| 27 | Guangzhou First People's Hospital |
| 28 | Guiqian International General Hospital |
| 29 | Hainan General Hospital |
| 30 | Hefei High-Tech Cardiovascular Hospital |
| 31 | Henan Chest Hospital |
| 32 | Henan Huazhong Fuwai Hospital |
| 33 | Henan Provincial People's Hospital |
| 34 | Huadong Hospital Affiliated to Fudan University |
| 35 | West China Hospital of Sichuan University (Huaxi Hospital) |
| 36 | Union Hospital, Tongji Medical College, Huazhong University of Science and Technology |
| 37 | Hubei Provincial People's Hospital |
| 38 | Hunan Provincial People's Hospital |
| 39 | Jiangxi Provincial People's Hospital |
| 40 | The First Hospital of Jilin University |
| 41 | Luoyang Central Hospital |
| 42 | Meizhou People's Hospital |
| 43 | Mianyang Central Hospital |
| 44 | Beijing Hospital (Ministry of Health) |
| 45 | The Second Affiliated Hospital of Nanchang University |
| 46 | Nanfang Hospital, Southern Medical University |
| 47 | Nanjing Drum Tower Hospital |
| 48 | Nanjing Municipal First Hospital |
| 49 | Ningbo First Hospital |
| 50 | Ningxia People's Hospital |
| 51 | Peking University First Hospital |
| 52 | Peking University Shenzhen Hospital |
| 53 | Qingdao Municipal Hospital |
| 54 | The Affiliated Hospital of Qingdao University |
| 55 | Qilu Hospital of Shandong University |
| 56 | Shanghai Changhai Hospital |
| 57 | Shanghai Chest Hospital |
| 58 | Shanghai Delta Health Hospital |
| 59 | Shanghai East Hospital |
| 60 | Shanghai General Hospital |
| 61 | Shanghai Geriatric Medical Center |
| 62 | Shanghai Renji Hospital |
| 63 | Shanghai Ruijin Hospital |
| 64 | Shanghai Xinhua Hospital |
| 65 | Zhongshan Hospital, Fudan University (Shanghai Zhongshan Hospital) |
| 66 | Shantou Central Hospital |
| 67 | Jincheng People's Hospital, Shanxi Province |
| 68 | Shenyang Military General Hospital |
| 69 | Shenzhen Fuwai Hospital |
| 70 | Shenzhen Hospital of the University of Hong Kong |
| 71 | Sichuan Provincial People's Hospital |
| 72 | Sun Yat-sen Memorial Hospital, Sun Yat-sen University |
| 73 | The Affiliated Hospital of Guangdong Medical University |
| 74 | The Affiliated Hospital of Hangzhou Normal University |
| 75 | The Second Affiliated Hospital of Harbin Medical University |
| 76 | The Fourth Affiliated Hospital of Harbin Medical University |
| 77 | The Eighth Affiliated Hospital of Sun Yat-sen University |
| 78 | The First Affiliated Hospital of Nanchang University |
| 79 | The First Affiliated Hospital of Wenzhou Medical University |
| 80 | The First Affiliated Hospital of Xi'an Jiaotong University |
| 81 | The First Affiliated Hospital of Guangdong Pharmaceutical University |
| 82 | The First Hospital of Lanzhou University |
| 83 | The First People's Hospital of Changzhou |
| 84 | The First People's Hospital of Nanning |
| 85 | Guangxi Zhuang Autonomous Region People's Hospital |
| 86 | The Second Affiliated Hospital of Nanchang University |
| 87 | Tianjin Chest Hospital |
| 88 | Tianjin Medical University General Hospital |
| 89 | Tianjin People's Hospital |
| 90 | Wuhan Asia Heart Hospital |
| 91 | Xiamen Cardiovascular Hospital, Xiamen University |
| 92 | Xijing Hospital |
| 93 | Xinjiang Cardiovascular and Cerebrovascular Disease Hospital |
| 94 | The First Affiliated Hospital of Xinjiang Medical University |
| 95 | Xinjiang People's Hospital |
| 96 | Yan'an Hospital of Kunming City |
| 97 | Yantai Yuhuangding Hospital |
| 98 | The First People's Hospital of Yulin |
| 99 | Yunnan Fuwai Hospital |
| 100 | Yunnan Second People's Hospital |
| 101 | The First Affiliated Hospital of Zhejiang University (Zhejiang No.1 Hospital) |
| 102 | The Second Affiliated Hospital of Zhejiang University (Zhejiang No.2 Hospital) |
| 103 | Zhejiang Run Run Shaw Hospital |
| 104 | Zhejiang Shaoyifu Hospital (Another name for Zhejiang Run Run Shaw Hospital) |
| 105 | Taizhou Hospital, Zhejiang Province |
| 106 | Zhengzhou No.7 Hospital |
| 107 | Zhengzhou Cardiovascular Hospital (Zhengzhou Seventh People's Hospital) |
| 108 | The First Affiliated Hospital of Zhengzhou University |
| 109 | The Second Affiliated Hospital of Zhengzhou University |
| 110 | The Fourth Affiliated Hospital of Zhengzhou University |
| 111 | The Fifth Affiliated Hospital of Zhengzhou University |
| 112 | The Seventh Affiliated Hospital of Zhengzhou University |
| 113 | The Eighth Affiliated Hospital of Zhengzhou University |
| 114 | The Tenth Affiliated Hospital of Zhengzhou University |
| 115 | The Eleventh Affiliated Hospital of Zhengzhou University |
| 116 | The Twelfth Affiliated Hospital of Zhengzhou University |
| 117 | The Thirteenth Affiliated Hospital of Zhengzhou University |
| 118 | The Fourteenth Affiliated Hospital of Zhengzhou University |
| 119 | Xiangya Second Hospital, Central South University |
| 120 | Zhongshan Hospital, Fudan University (Xiamen Branch) |
| 121 | Zhongshan People's Hospital |
| 122 | Zhoukou Central Hospital |
| 123 | Zhuhai People's Hospital |

**Supplemental Table 2**. Missing rates of study variables and management of missingness.

| Variables | Missing, N (%) | Management of missing data |
| --- | --- | --- |
| Annulus area | 77 (4.69%) | Imputed using MissForest, a random forest imputation algorithm for missing data implemented in the R software |
| Height of left coronary artery | 53 (3.23%) |  |
| Height of right coronary artery | 54 (3.29%) |  |
| Annulus diameter | 342 (20.8%) |  |
| LVEF | 988 (60.2%) | Not imputed |

Abbreviations: LVEF, left ventricular ejection fraction.

**Supplemental Table 3.** Patients’ baseline and procedural characteristics across BAV morphologies.

|  | Total BAV cohort | BAV type 0 | BAV type 1 | BAV type 2 | *p* value |
| --- | --- | --- | --- | --- | --- |
|  | (n = 920) | (n = 394) | (n = 510) | (n = 16) |  |
| Baseline characteristics | | | | | |
| Age (years) | 70.6 ± 7.79 | 69.6 ± 7.68 | 71.5 ± 7.59 | 65.4 ± 11.5 | <0.001 |
| Male (n, %) | 548 (59.6) | 199 (50.5) | 338 (66.3) | 11 (68.8) | <0.001 |
| LVEF (%)* | | | | | 0.960 |
|  | 55.8 ± 12.6 | 56.0 ± 13.0 | 55.7 ± 12.4 | 55.0 ± 12.6 |  |
| Annulus area (mm^2^) | | | | | <0.001 |
|  | 484.0 (422.0, 562.0) | 455.5.0 (400.0, 539.0) | 500.0 (436.0, 576.0) | 533.0 (479.8, 651.5) |  |
| Annulus diameter (mm) | | | | | 0.002 |
|  | 40.8 ± 6.94 | 41.7 ± 6.87 | 40.2 ± 6.98 | 38.0 ± 4.28 |  |
| Height of coronary artery (mm) | | | | | |
| Left coronary artery | 14.7 ± 3.32 | 16.1 ± 3.35 | 13.6 ± 2.85 | 14.7 ± 3.07 | <0.001 |
| Right coronary artery | 16.8 ± 3.24 | 17.4 ± 3.30 | 16.3 ± 3.12 | 17.4 ± 3.25 | <0.001 |
| Annulus aneurysm (n, %) | | | | | 0.610 |
|  | 53 (5.80) | 23 (5.80) | 30 (5.90) | 0 |  |
| Annulus calcification (n, %) | | | | | 0.340 |
| None | 638 (69.3) | 264 (67.0) | 363 (71.2) | 11 (68.8) |  |
| Mild | 175 (19.0) | 85 (21.6) | 89 (17.5) | 1 (6.20) |  |
| Moderate | 71 (7.70) | 30 (7.60) | 38 (7.50) | 3 (18.8) |  |
| Severe | 36 (3.90) | 15 (3.80) | 20 (3.90) | 1 (6.20) |  |
| LVOT calcification (n, %) | | | | | 0.660 |
| None | 779 (84.7) | 329 (83.5) | 437 (85.7) | 13 (81.2) |  |
| Mild | 91 (9.90) | 42 (10.7) | 48 (9.40) | 1 (6.20) |  |
| Moderate | 39 (4.20) | 17 (4.30) | 20 (3.90) | 2 (12.5) |  |
| Severe | 11 (1.20) | 6 (1.50) | 5 (1.00) | 0 |  |
| Leaflet calcification (n, %) | | | | | 0.012 |
| None | 105 (11.4) | 54 (13.7) | 50 (9.80) | 1 (6.20) |  |
| Mild | 138 (15.0) | 53 (13.5) | 83 (16.3) | 2 (12.5) |  |
| Moderate | 305 (33.2) | 115 (29.2) | 188 (36.9) | 2 (12.5) |  |
| Severe | 372 (40.4) | 172 (43.7) | 189 (37.1) | 11 (68.8) |  |
| Sizing of prosthesis (mm) | | | | | <0.001 |
| 20 | 66 (7.20) | 38 (9.60) | 28 (5.50) | 0 |  |
| 23 | 362 (39.3) | 182 (46.2) | 173 (33.9) | 7 (43.8) |  |
| 26 | 371 (40.3) | 136 (34.5) | 228 (44.7) | 7 (43.8) |  |
| 29 | 121 (13.2) | 38 (9.60) | 81 (15.9) | 2 (12.5) |  |
| Valvular deployment height (n, %)** | | | | | <0.001 |
| 100/0 | 209 (22.7) | 113 (28.7) | 95 (18.6) | 0 |  |
| 90/10 | 436 (47.4) | 201 (51.0) | 222 (43.5) | 0 |  |
| 80/20 | 248 (27.0) | 70 (17.8) | 176 (34.5) | 2 (12.5) |  |
| 70/30 | 19 (2.10) | 8 (2.00) | 11 (2.20) | 13 (81.2) |  |
| 60/40 | 8 (0.90) | 2 (0.50) | 6 (1.20) | 1 (6.20) |  |

*The LVEF statistics were based on 374 patients due to 546 cases with missing value for LVEF.

**The implantation height was expressed as the percentage of the stent lying on the aortic and the ventricular sides^26^.

Abbreviations: BAV, bicuspid aortic valve; LVEF, left ventricular ejection fraction; LVOT, left ventricular outflow tract; TAV, tricuspid aortic valve.
