# Supplementary figures and images for: Real-World Observations in the Treatment of Aortic Stenosis With the Transfemoral SAPIEN 3 Transcatheter Heart Valve: Insights From China
Source: Rev Cardiovasc Med. 2025 May 22;26(5):28800. doi: 10.31083/RCM28800 (PMC12135656; doi:10.31083/RCM28800)

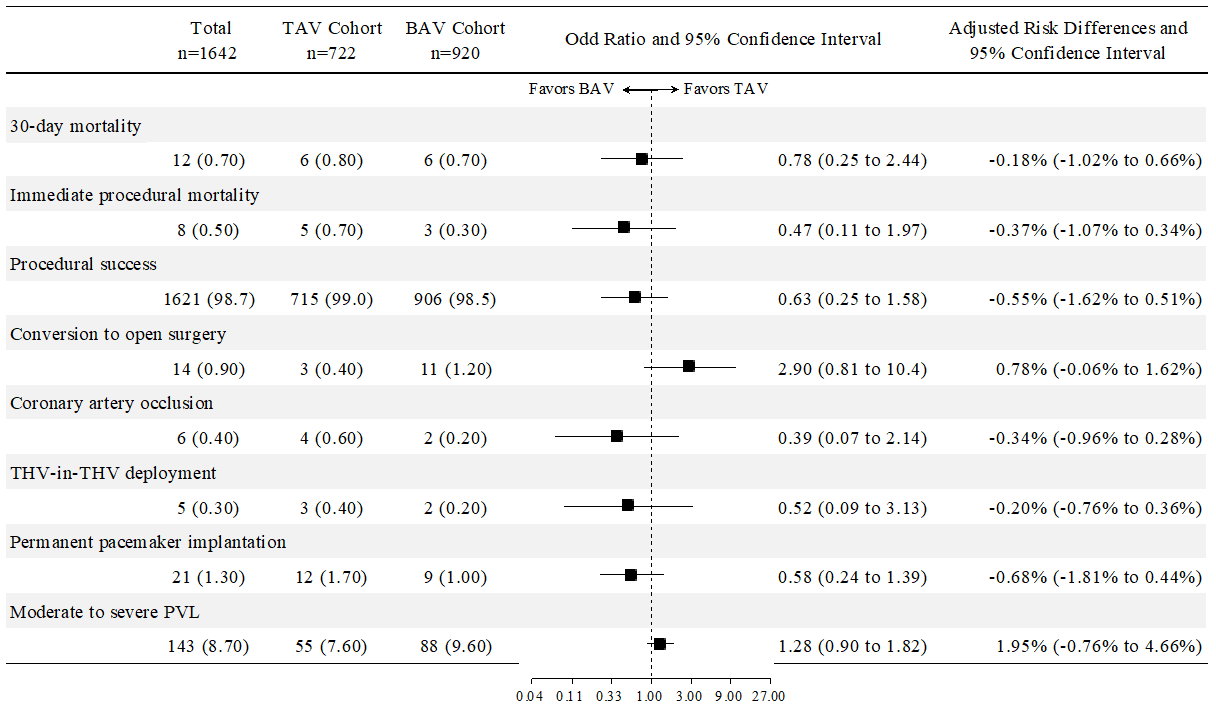

Supplement: Supplementary file 1 [file 2153-8174-26-5-28800-s1.zip › Supplementary Fig. 1.png]

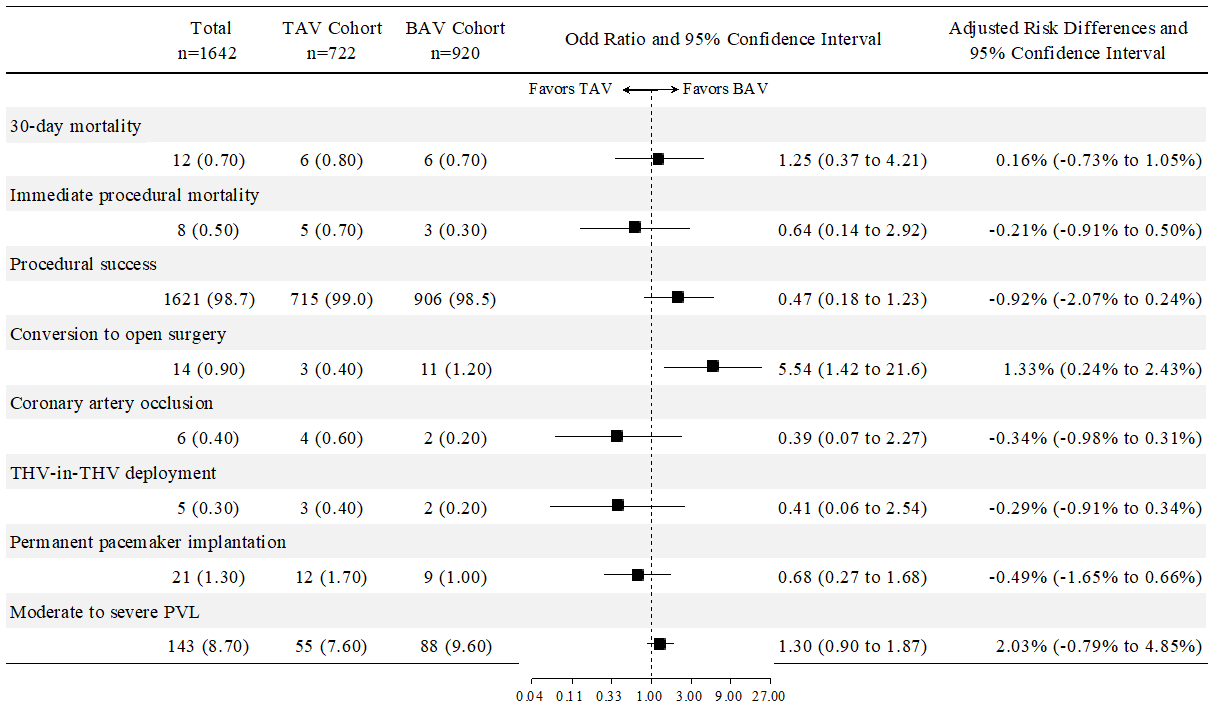

Supplement: Supplementary file 1 [file 2153-8174-26-5-28800-s1.zip › Supplementary Fig. 2.png]
